# In Vitro and In Silico Approaches to Generating and Identifying Angiotensin-Converting Enzyme I Inhibitory Peptides from Green Macroalga *Ulva lactuca*

**DOI:** 10.3390/md17040204

**Published:** 2019-03-30

**Authors:** Marco Garcia-Vaquero, Leticia Mora, Maria Hayes

**Affiliations:** 1 School of Veterinary Medicine, University College Dublin (UCD), Belfield, Dublin 4, Ireland; 2 Instituto de Agroquímica y Tecnología de Alimentos (CSIC), Avenue Agustín Escardino 7, 46980 Paterna (Valencia), Spain; lemoso@iata.csic.es; 3 Food Biosciences Department, TEAGASC, Food Research Centre, Ashtown, Dublin 15, Ireland; maria.hayes@teagasc.ie

**Keywords:** seaweed protein, protein hydrolysate, ACE-I, renin, allergenicity, in silico analysis, functional food, bioactive peptide, bioinformatics

## Abstract

A protein extract was generated from the macroalga *Ulva lactuca*, which was subsequently hydrolysed using the food-grade enzyme papain and angiotensin-converting Enzyme I and renin inhibitory peptides identified using a combination of enrichment strategies employing molecular weight cutoff filtration and mass spectrometry analysis. The generated hydrolysates with the most promising in vitro activity were further purified using preparative RP-HPLC and characterised. The 1 kDa hydrolysate (1 kDa-UFH), purified and collected by preparative RP-HPLC at minutes 41‒44 (Fr41‒44), displayed statistically higher ACE-I inhibitory activities ranging from 96.91% to 98.06%. A total of 48 novel peptides were identified from these four fractions by LC-MS/MS. A simulated gastrointestinal digestion of the identified peptide sequences was carried out using in silico enzyme cleavage simulation tools, resulting in 86 peptide sequences that were further assessed for their potential activity, toxicity and allergenicity using multiple predictive approaches. All the peptides obtained in this study were predicted to be non-toxic. However, 28 out of the 86 novel peptides released after the in silico gastrointestinal digestion were identified as potential allergens. The potential allergenicity of these peptides should be further explored to comply with the current labelling regulations in formulated food products containing *U. lactuca* protein hydrolysates.

## 1. Introduction

Cardiovascular disease (CVD) is the main cause of death in Europe [1], and among CVD, hypertension has become a problem of global, epidemic proportions [2]. Long-term hypertension results in increased risk of stroke, heart attack, arterial aneurysm and kidney failure [3,4]; even moderate hypertension can shorten the life span [3]. Hypertension is a global problem that influences the health of the population and also exerts a substantial influence on the economy. The direct medical costs associated with CVD in the United States in 2010 were valued at approximately 273 billion USD and this figure is estimated to reach 818 billion USD by 2030 [5].

The inhibition of enzymes such as renin (EC 3.4.23.15) and angiotensin-I-converting enzyme (ACE-I, EC 3.4.15.1) is known to be effective at lowering blood pressure [6]. These enzymes operate within a number of metabolic pathways including the renin‒angiotensin‒aldosterone system (RAAS), the renin‒chymase system (RCS), the kinin‒nitric oxide system (KNOS) and the neutral endopeptidase system (NEPS) [7]. Synthetic drugs that target renin and ACE-I are available for the clinical treatment of hypertension. These drugs include captopril, enalapril and lisinopril. Despite the efficiency of these drugs, adverse side effects including dry cough, taste disturbances, skin rashes, renal failure, congenital malformations and angioneurotic oedema have been reported [8,9]. Thus, it is necessary to find safer enzyme inhibitors for the prevention and remedy of hypertension from natural products [10].

Bioactive peptides or cryptides are sequences of between 2 and 30 amino acids. These peptides show no activity within the parent protein, but after their release by different methods, i.e., enzymatic hydrolysis or fermentation, they may show beneficial biological activities. Thus, bioactive peptides obtained from different food protein sources are considered a promising alternative to synthetic drugs [2] and these food-based strategies to control high blood pressure can be considered an excellent intervention to improve health and wellness and decrease health care costs [3]. Numerous renin and ACE-I-inhibitory peptides have been obtained previously by hydrolysis of animal proteins from milk [11], eggs [12] and blood [6,13]. Several peptides were also identified from the macroalga *Palmaria palmata* [14] and indeed terrestrial crops [15,16].

Macroalgae are a rich source of multiple biologically active compounds including polysaccharides and proteins [17,18]. Chlorophyta (green) and Rhodophyta (red) macroalgae are the most promising algae to be exploited as sources of protein for functional food and feed development [17,19]. Amongst the green macroalgae, *Ulva* sp. is a promising biomass as it has a worldwide distribution [20] and levels of protein documented in the literature of between 7% and 33% of the dry weight of the algal plant [21,22]. Apart from its total protein content, marine organisms have adapted to extreme environmental conditions including high salt concentration and pressure conditions and the composition and primary sequences of amino acids of these marine proteins are different from those of land proteins [10]. Thus, marine macroalgae are interesting protein resources for the generation of novel antihypertensive (renin and ACE-I inhibitory) peptides by enzymatic hydrolysis [10].

The aim of this study was to generate and characterise novel bioactive protein hydrolysates from *U. lactuca* as this macroalgae is considered a relatively untapped source of biologically active molecules. The protein extracted from *Ulva* sp. was hydrolysed using the food-grade enzyme papain; enriched using 1, 3 and 10 kDa molecular weight cutoff (MWCO) filtration membranes; and further purified using preparative RP-HPLC. The antihypertensive activities of the fractions was measured in vitro for renin and ACE-I inhibition and active fractions subsequently characterised using mass spectrometry (LC/MS/MS). The amino acid sequence of peptides contained in these fractions was determined and they were subsequently assessed for their bitterness, resistance to gastrointestinal digestion, potential toxicity and estimated allergenicity using multiple in silico predictive tools.

## 2. Results and Discussion

**Figure 1:**
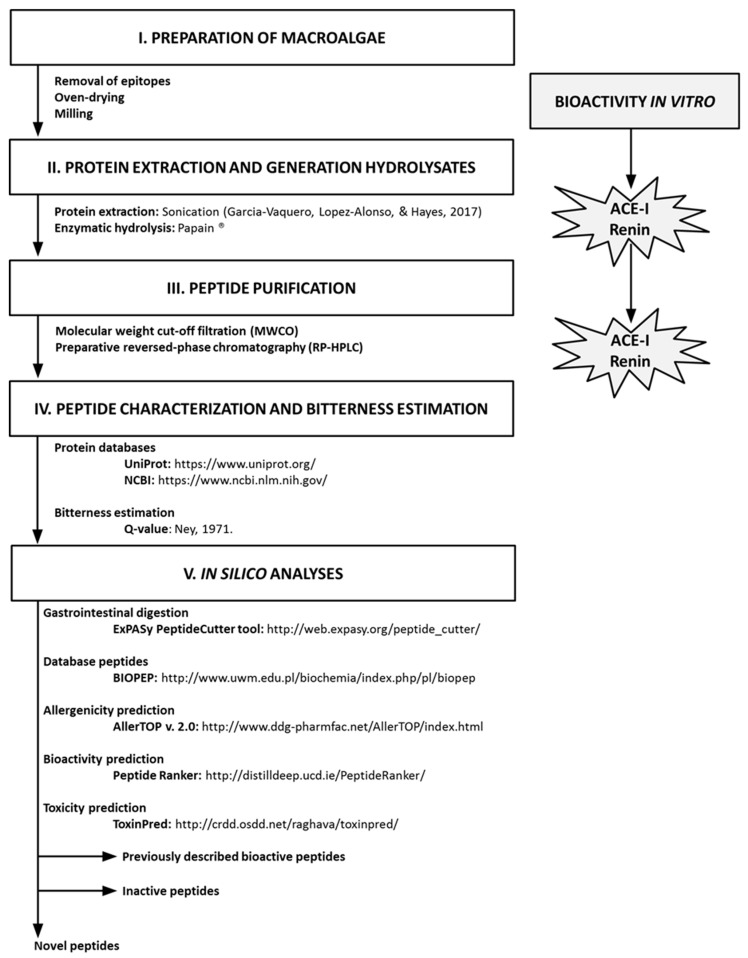
Schematic representation of the procedures used to generate and identify bioactive peptides from *Ulva lactuca* using in vitro and in silico tools.

The methodological approaches used in this study to generate and identify bioactive peptides from *Ulva* sp. are schematically represented in Figure 1 and further described in Section 3.

### 2.1. Protein Extraction

The extracts generated from *Ulva* sp. had a protein content of 69.19 ± 1.44%, as assessed by the BCA method. Previous studies extracting protein from macroalgae *Himanthalia elongata* using a sonication water bath also reported similar protein contents (63.38 ± 0.49%) in the algal extracts [23]. Moreover, the yields of total protein extracted from *Ulva* sp. were 4649.98 ± 96.68 mg of protein per 100 g of dried biomass.

### 2.2. Generation of Hydrolysates and Antihypertensive Activities In Vitro

A papain hydrolysate of the crude *Ulva* protein was generated in order to release biologically active peptides or cryptides from the parent proteins. Previous reports emphasised the need to generate and use protein hydrolysates as the presence of intact or partially hydrolysed proteins can induce immune-mediated allergic reactions in sensitive individuals [24]. Although the need for amino acids could also be supplied by mixtures of synthetic amino acids, the generation of protein hydrolysates remains the most promising source of peptides [24]. The protein hydrolysates can be produced at a large scale industrially, and the peptides generated have good absorption and stability when compared to free amino acids, particularly glutamine, tyrosine and cysteine [24].

To concentrate and purify the different peptides generated during the enzymatic hydrolysis, the full hydrolysate was filtered through MWCO filtration units of 1, 3, and 10 kDa separately, generating three additional ultra-filtered fractions, namely 1 kDa-UFH, 3 kDa-UFH and 10 kDa-UFH. The control of the molecular size of the peptides generated after a protein hydrolysis is an essential step in the development of dietary products [24]. MWCO filtration has been proven to be the most efficient post-hydrolysis procedure to separate non-hydrolysed proteins, high MW peptides or residues of the proteolytic enzymes added to perform the hydrolysis [24].

**Figure 2:**
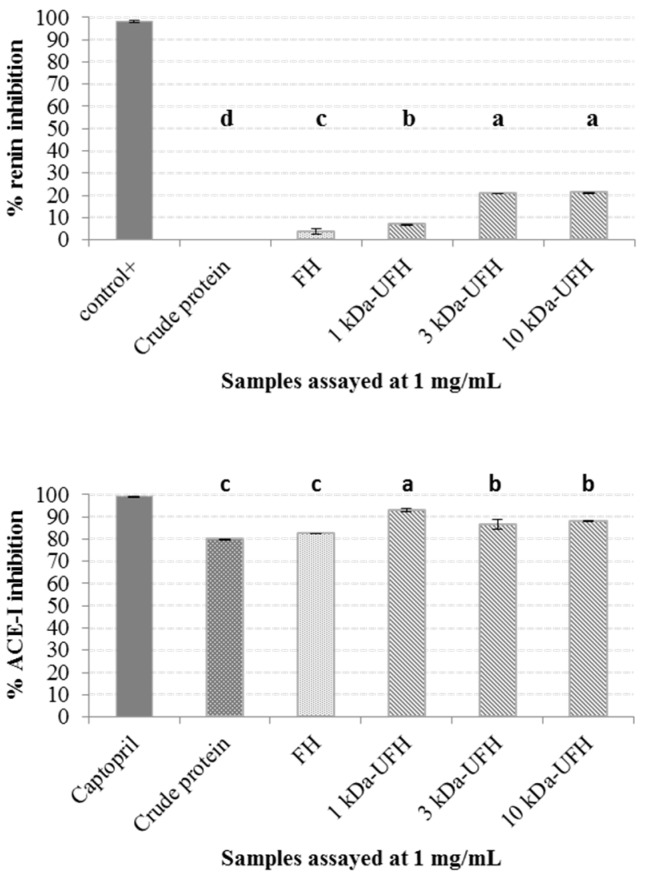
In vitro renin and angiotensin-I-converting enzyme (ACE-I) inhibitory activities of crude protein, full hydrolysate (FH) and ultra-filtered hydrolysates (1, 3 and 10 kDa-UFH) generated from *Ulva* sp. The samples and positive controls (the renin inhibitor Z-Arg-Arg-Pro-Phe-His-Sta-Ile-His-Lys-(Boc)-OMe and the ACE-I inhibitor captopril) were assayed at 1 mg/mL. The results are expressed as mean ± standard deviation of the mean (SEM). The different letters in the figure indicate statistical significant differences (*P* < 0.05) between the different samples tested.

The in vitro renin and ACE-I inhibitory activities of crude protein, full hydrolysate (FH) and the three ultra-filtered fractions (1 kDa-UFH, 3 kDa-UFH and 10 kDa-UFH) are shown in Figure 2. When assayed for renin inhibition, the crude protein did not inhibit renin and the full hydrolysate inhibited renin by 3.70 ± 1.39% compared to the specific renin inhibitor, Z-Arg-Arg-Pro-Phe-His-Sta-Ile-His-Lys-(Boc)-OMe, which was used as the positive control. Ultrafiltration enhanced renin inhibitory activity and the different fractions inhibited renin as follows: 3 kDa by 20.79 ± 0.15%; 10 kDa-UFH by 21.19 ± 0.27% and 1 kDa-UFH by 6.89 ± 0.18%. The renin inhibitory activities were all less than 20% and compared negatively to previously identified renin inhibitors such as hydrolysates from the macroalga *Palmaria palmata* [14] or other terrestrial crops such as *Avena sativa* [25].

In the case of ACE-I, crude *Ulva* sp. protein inhibited ACE-I by 79.87 ± 0.18% at a concentration of 1 mg/mL and after hydrolysis with papain the ACE-I inhibition activity of the FH was increased to 82.37 ± 0.05% when assayed at 1 mg/mL concentrations compared to the initial protein. Following ultrafiltration, ACE-I inhibition also increased, with the maximum inhibitory activity observed for the 1 kDa-UFH (93.03 ± 0.87%) followed by both the 3 kDa-UFH (86.64 ± 2.17%) and 10 kDa-UFH (88.12 ± 0.02%) when all fractions were assayed at a concentration of 1 mg/mL. These results are in agreement with previous reports that describe the structure of peptides with ACE-I inhibitory activities as short amino acid sequences, including di and tri-peptides [26] or even up to 10 amino acid residues [27]. The low concentration of small peptides in the FH compared to the ultra-filtered fractions and the blocking effect of large peptides on the antihypertensive activity of small peptides, could explain these results. Moreover, the ACE-I inhibitory activities of all the fractions, particularly the 1 kDa-UFH, were higher than other promising peptide fractions obtained from terrestrial plants and animal proteins. Gangopadhyay, Wynne, O’Connor, Gallagher, Brunton, Rai and Hayes [15] generated protein hydrolysates from barley (*Hordeum vulgare*) and purified the hydrolysate using membranes, being the most promising fractions the 3 kDa fraction that inhibited ACE-I by 70.37 ± 0.67%, followed by the 10 kDa UFH (57.42 ± 4.68%) and the full hydrolysate (47.23 ± 2.62%). Similarly to our results, Lafarga, Rai, O’connor and Hayes [6] generated hydrolysates from bovine haemoglobin proteins and the highest ACE-I inhibition was appreciated in the peptides contained in the 1 kDa fraction that showed approximately 40% of ACE-I inhibition, compared to the 3 and 10 kDa samples.

### 2.3. Purification by Preparative Reversed-Phase High-Performance Liquid Chromatography (RP-HPLC) and Antihypertensive Activity In Vitro

**Figure 3:**
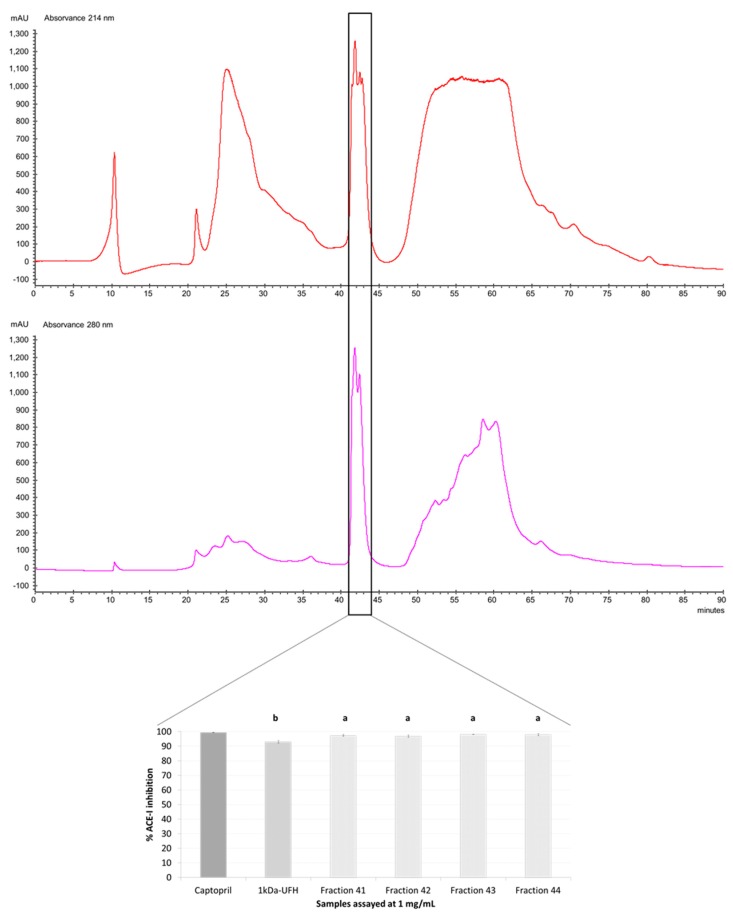
Preparative reversed-phase HPLC (RP-HPLC) chromatogram obtained from the 1 kDa-UFH monitored at wavelengths 214 and 280 nm. The ACE-I inhibitory activities of captopril, 1 kDa-UFH and the samples from RP-HPLC collected at minutes 41‒44 (Fr41‒Fr44) are also presented at the end of the figure. The results are expressed as mean ± standard deviation of the mean (SEM). The different letters in the figure indicate statistical significant differences (*P* < 0.05) between the samples tested.

To further purify the most promising peptides contained in the 1 kDa-UFH fraction, preparative RP-HPLC was performed, the peptides were collected every minute and the separation was monitored at 214 nm, wavelength of absorbance of peptide bonds, and 280 nm, which is the wavelength of choice to detect residues of aromatic amino acids [28]. The RP-HPLC chromatogram obtained for the 1 kDa-UFH fraction of *Ulva* sp. is presented in Figure 3. The chromatogram at 214 nm shows main absorbance peaks for peptide bonds at minutes 9‒11, 21‒30, 41‒44 and a final plateau at 52‒62 min. However, when monitoring the absorbance at 280 nm, the main peaks related to the presence of aromatic amino acids were mainly appreciated at minutes 41‒44 and the previously observed plateau at minutes 52‒62 decreased significantly. The presence of branched amino acids at the N-terminal position and aromatic amino acid residues at the C-terminal position in peptides has been described as one key structural feature governing the ACE-I inhibitory activity of peptides [29]. Thus, peptides containing amino acids such as leucine, valine, alanine and the aromatic amino acids tyrosine, phenylalanine or tryptophan are likely to inhibit ACE by competing for binding to the catalytic sites of the enzyme [30,31]. From the chromatogram shown in Figure 3, using RP-HPLC monitored at 280 nm, the fractions collected at minutes 41‒44 were likely to content high amounts of aromatic amino acids and, thus, have a strong potential to inhibit ACE-I. When the peptide fractions 41‒44 were assayed at 1 mg/mL for ACE-I inhibition, the antihypertensive activity of all these fractions was statistically higher compared to the previously assayed 1 kDa-UFH. The average ACE-I of all the fractions was in all cases higher than 95%, close to the levels of inhibition of ACE-I of the captopril used as a positive control. The peptides contained in these four peptide fractions (Fr41‒44) were identified by HPLC-MS/MS.

### 2.4. Identification of Peptides Using HPLC-MS/MS and Bitterness Estimation

**Figure 4:**
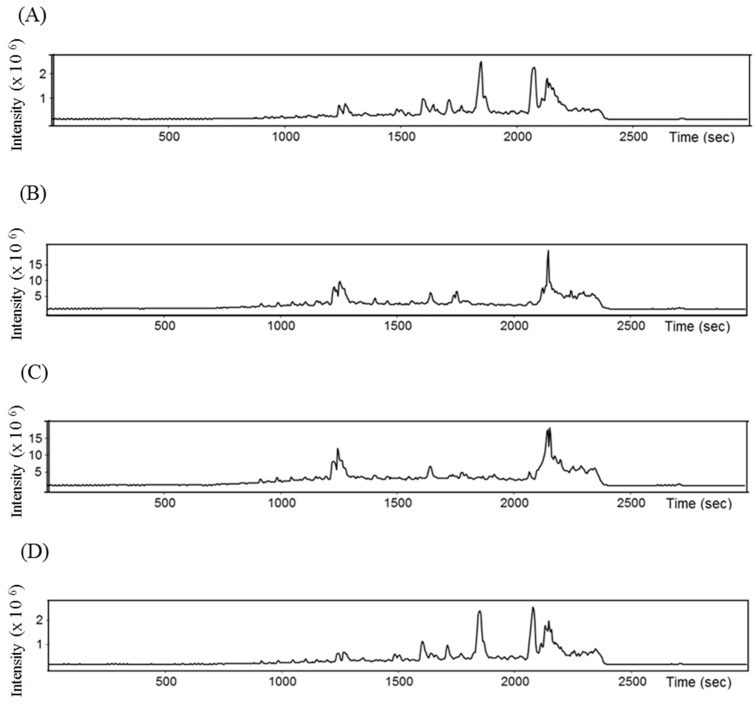
Total ion chromatogram (TIC) separation of the selected HPLC fractions for mass spectrometry analysis. (**A**) corresponds to Fr41, (**B**) to Fr42, (**C**) to Fr43 and (**D**) to Fr44.

A total of 48 peptides were identified from the four selected fractions (Fr41‒44) of 1 kDa-UFH showing potent ACE-I inhibitory activities. The amino sequences of these novel peptides and their modifications were further characterized by the observed and calculated molecular masses, theoretical and observed mass/charge (*m*/*z*), and charge (z) as it is shown in Table 1. The total ion chromatogram after the HPLC separation in mass spectrometry is also presented in Figure 4. The amino acid sequences had green algae origin and were compared to previously reported peptides in the BIOPEP database [32]. All the peptides identified were novel and not previously reported in this database. As these peptides are intended for oral intake, one factor that could influence consumption when incorporated into a food product could be the taste [24]. The process of protein hydrolysis generates short peptides that are normally bitter and not accepted by Western consumers, regardless of the potential health benefits of the products [33]. The Q-values were calculated for the identified peptides based on the method of Ney [34], who estimates the bitterness of the peptides on the basis of the solubility data of each individual amino acid. When a peptide has a molecular weight (MW) lower than 6 kDa and the Q-value of the peptide exceeds 1400 cal/mol, the sequence is predicted to be bitter. The peptides identified in Fr41‒44 have generally Q-values below the threshold of bitterness (see Table 1). Only the sequences SAGVLPWK, GAAPTPPSPPPATKPSTPPKPPT, IECCLLFALV, PVGCLPK, DAVEIWRVK, DEVIPGAL, PKPPALCN and PPNPPNPPN with Q-values ranging from 1440 to 1743.33 cal/mol could be predicted to be bitter. Although this estimation method can be applied to the majority of the known peptides, a few authors have also suggested that the bitterness of the peptides could be related to the MW of the amino acid sequences rather than the Q-value [35].

### 2.5. In Silico Analyses

#### 2.5.1. In Silico GI Enzymatic Digestion

Several issues that will influence the future applicability and usage of bioactive peptides include the water solubility of the molecules, the stability of the products and the bioavailability of the peptides when passing through biological barriers such as the GI tract [6]. The resistance of peptides to the enzymatic degradation in the GI tract will determine the bioavailability of the initial peptides. Moreover, novel amino acid sequences could be released from the parent peptide during the process of digestion and may exert toxic, allergenic or other potent biological activities when absorbed. After the in silico enzymatic simulations performed, only a few peptides remained intact, namely ATKPAN, SGAASASGAA (from Fr41), AGGPNQPPN, AANITVPAAN (identified in Fr42), EAEPAEAA, GAAPTPPSPPPATKPSTPPKPPT (obtained in Fr43) and PPNPPNPPN (Fr44). All the peptides obtained from the in silico enzymatic simulation were further compared with the BIOPEP database [32] to identify previously reported bioactive peptides. There were 16 di- and tri-peptides released after the GI enzymatic simulation previously reported as bioactives (see Table 2).

The main biological activities described for these amino acid sequences are antihypertensive (ACE-I inhibitors) and anti-diabetic (dipeptidyl peptidase IV inhibitors) peptides. Furthermore, the sequence GGV was described as an inhibitor of the enzyme 3-hydroxy-3-methyl-glutaryl-CoA reductase (HMG-CoA reductase) and could have a hypocholesterolemic effect [36].

#### 2.5.2. In Silico Prediction of Allergenicity and Bioactivity

Food allergy is a huge concern in the development of nutraceuticals and the formulation of novel and safe food products [42]. Allergic reactions start when immunoglobulin E binds and activates mast and basophil cells upon the contact with food allergens. These activated cells release granules containing inflammatory mediators and other molecules, generating an immediate allergic reaction that could be life-threatening [43]. Immunoglobulin E-mediated food allergies affect between 3% and 8% of children and 1–3% of adults in developed countries and their prevalence and severity are increasing. The most commonly described sources of food allergens are milk, eggs and wheat, which produce reactions that are normally overcome with age as the patients acquire tolerance. However, peanuts, tree nuts and fish allergies often persist over a lifetime [43]. The potential allergenicity of proteins from macroalgae has not yet been fully explored. Recently, Polikovsky, Fernand, Sack, Frey, Müller and Golberg [42] reported the first study on the existence of potential allergens in proteins extracted from *Ulva* sp. The authors suggested that the potential allergenicity of *Ulva* sp. proteins will depend on the extraction conditions as the protocols of extraction will influence the mass transfer of molecules from the macroalgal biomass to the extract. Moreover, the European Food Safety Authority (EFSA) favours the use of in silico tools to predict the potential allergenicity of food proteins [44].

In the current study, the 70 novel peptides released after the in silico GI enzymatic digestion were further analysed for their potential allergenicity in silico using AllerTop version 2.0 (http://www.ddg-pharmfac.net/AllerTOP). This in silico prediction tool is based on the transformation of amino acid descriptors of the protein strings into uniform vectors, followed by using the k nearest neighbours algorithm (kNN, *k* = 1) to classify the peptides based on a set containing 2427 known allergens and 2427 non-allergens [45]. The amino acid sequences that could be described as probably allergenic are summarised in Table 3, together with their nearest allergenic protein, while the probably non-allergenic 42 peptides are summarised in Table 4.

This information will be useful to prevent allergic systemic reactions produced when the allergens cross the intestinal mucosa and enter the circulation, creating a reaction that may compromise the circulatory and nervous systems [43]. The European Food Safety Authority (EFSA) favours the use of in silico tools to predict the initial potential allergenicity of food proteins [44]. The results on the allergenicity of these peptides suggest that papain hydrolysates from *Ulva* sp. could result in potentially allergenic peptides after GI digestion and, thus, this parameter should be further assessed in vitro and in vivo to comply with the current food allergen labelling European regulations such as the Regulation (EU) No.1169/2011.

The potential of the allergenic and non-allergenic peptides to be bioactive is also summarised in Table 3 and Table 4, as analysed using PeptideRanker. Several peptides scored high in peptide ranker (>0.6); in particular, peptides GPPPPSP and GTF, with scores over 0.8, show potential for further in vivo evaluation as some of the peptides assayed in vitro did not perform as well as expected using in vivo models [46].

#### 2.5.3. In Silico Prediction of Toxicity

The protein hydrolysates of this study were generated from edible parts of *Ulva* sp. destined for human consumption and the enzyme papain is a food-grade molecule obtained from plant (*Carica papaya*). Moreover, protein hydrolysates generated using similar enzymatic procedures from animal [6,13] and other edible protein sources such as plants [15] and algae [14] have not been reported to pose a serious health risk to consumers. None of the identified peptides in this study were predicted to be toxic using ToxinPred (http://crdd.osdd.net/raghava/toxinpred/) as seen in the negative SVM scored summarised in Table 3 and Table 4. The toxicity assessments should be further explored using cell lines and animal studies before the product is made available for human consumption. However, protein hydrolysates and low MW peptides are generally considered non-toxic [6] and are less allergenic than full or partially hydrolysed proteins [24]. Protein hydrolysates have been used to develop hypoallergenic infant formulas [47], but also in specific diets to treat patients with metabolic disorders affecting amino acid digestion, absorption and metabolism [24]. Other uses of protein hydrolysates include the treatment of malnutrition associated with age [48] or other diseases such as cancer, trauma, burns and hepatic encephalopathies, mainly due to the easy absorption of short peptides [24].

## 3. Material and Methods

### 3.1. Biological Materials

The macroalgae *Ulva lactuca* was kindly supplied by Portomuiños (Galicia, Spain). Macroalgae were collected at Muxía (A Coruña, Galicia, Spain) on 10 June 2013. Following harvest, the seaweeds were cleaned and epitopes removed, oven-dried, milled and vacuum preserved until further analysis.

### 3.2. Chemicals

Ammonium sulphate, formic acid (FA), trifluoroacetic acid (TFA), acetonitrile (ACN), HPLC-grade water CHROMASOLV^®^, dimethyl sulfoxide (DMSO), papain (EC 3.4.22.2) from *Carica papaya* ≥3 U/mg, QuantiPro BCA assay kit and the inhibitors of ACE-I (Captopril©) and renin (Z-Arg-Arg-Pro-Phe-His-Sta-Ile-His-Lys-(Boc)-OMe) were all supplied by Sigma-Aldrich (Dublin, Ireland). The ACE-I and renin inhibitory screening assay kits were supplied by Dojindo Laboratories (Kumamoto, Japan) and Cayman Chemical Company (Ann Arbor, MI, USA), respectively. All the other chemicals used were of analytical grade.

### 3.3. Protein Extraction and Quantification

Crude protein was extracted following the method previously described by Garcia-Vaquero, Lopez-Alonso and Hayes [23]. Briefly, 10 g of dried seaweed were suspended in 1 L of ultrapure water and ultra-sonicated for 1 h (Branson 3510EMT, Branson Ultrasonics Corporation, Danbury, CT, USA) and left overnight on a magnetic stirrer plate (IKA RCT basic safety control) at 4 °C. The solution was then centrifuged at 10,000× *g* for 1 h and the supernatant decanted. The pellet fraction was re-suspended in 0.5 L of ultrapure water and subjected to a second extraction procedure as described above. Supernatants from both days were pooled together; the solution was then saturated to 80% with ammonium sulphate for 1 h at 4 °C and centrifuged at 20,000× *g* for 1 h to precipitate the protein. The protein precipitates were subsequently dialyzed using Thermo Scientific™ SnakeSkin™ dialysis tubing, 3.5 kDa MWCO (Thermo Fisher Scientific, Hudson, NH, USA) against ultrapure water at 4 °C. Dialyzed protein extracts were freeze-dried and stored at ‒20 °C until further analysis.

The protein contents in the crude extracts of *Ulva lactuca* were determined using the QuantiPro BCA Assay Kit (Sigma-Aldrich, Saint Louis, MO, USA). Samples were prepared according to the manufacturer’s instructions using Greiner 96-well flat-bottom plates (Sigma) and absorbance values were read at 562 nm in a microplate reader (FLUOstar Omega, BMG Labtech, Offenburg, Germany).

### 3.4. Protein Hydrolysis and Molecular Weight Cutoff Filtration

*Ulva* sp. hydrolysates of the crude protein extracts were prepared using a 1 L bioreactor with temperature and pH control (Bioflo 110, New Brunswick Scientific Co Inc., Edison, NJ, USA). The crude protein was dispersed in ultrapure water at a concentration of 0.01 g/mL at a total volume of 0.5 L. The hydrolysis was carried out by the addition of 1% papain^®^. Conditions used included stirring at 300 rpm, pH 6 and temperature maintained at 60 °C for a 24 h period. The hydrolysis was stopped by heating the mixture to 95 °C for 10 min in a water bath. The full hydrolysate (FH) was further concentrated using 10 kDa, 3 kDa and 1 kDa MWCO membranes obtaining three fractions, namely 1 kDa-UFH, 3 kDa-UFH and 10 kDa-UFH, respectively. All the hydrolysates were freeze dried, vacuum-packed and stored at ‒20 °C until further use.

### 3.5. Preparative Reversed-Phase High-Performance Liquid Chromatography (RP-HPLC)

The 1 KDa-UFH was further purified by preparative RP-HPLC (Varian Pro-Star, Agilent Technologies, USA) using a Luna^®^ C18 (Phenomenex^®^, 5 µm 100 μm × 21.2 mm). Prior to analysis, samples were re-dissolved (5% *w*/*v*) in HPLC-grade water with 0.1% TFA and filtered through 0.2 μm filters (Agilent Captiva, Agilent Technologies, Santa Clara, CA, USA). The column was equilibrated with HPLC-grade water with 0.1% TFA at a flow rate of 1 mL/min for 10 min. Following the injection (1 mL) the sample was separated using a linear gradient of ACN (2–98% *v*/*v*, 90 min) containing 0.1% TFA at a flow rate of 1 mL/min. The absorbance was monitored at wavelengths 214 and 280 nm and the eluted fractions were collected every minute. The solvents contained in the fractions (ACN and TFA) were vapored under nitrogen and the peptide fractions were freeze-dried.

### 3.6. In Vitro Antihypertensive Activities

#### 3.6.1. ACE-I Inhibition Assay

The ACE-I inhibitory activity of the samples was measured according to a method based on the detection of the amount of 3-hydroxybutyrate (3-HB) generated from 3-hydroxybutyryl-Gly-Gly-Gly (3HB-GGG). The measurement was done using the materials enclosed in an ACE-I inhibition kit-WST (Dojindo Laboratories) and prepared according to the manufacturer’s instructions. All fractions were assayed at a concentration of 1 mg/mL in HPLC-grade water in triplicate and standard deviations of the mean (SEM) were calculated. Briefly, the enzyme working solution was prepared by dissolving enzyme B in 2 mL of HPLC-grade water and adding 1.5 mL of this solution to enzyme A. The indicator working solution was prepared by dissolving enzyme C and coenzyme with 3 mL of HPLC-grade water each and adding 2.8 mL of each of them to the indicator solution to prepare the indicator working solution. Negative control or blank 1 was prepared by adding 20 μL of HPLC-grade water and 20 μL of substrate buffer. Reagent blank 2 wells were prepared by adding 40 μL of HPLC-grade water and 20 μL of substrate buffer. Inhibitor wells were prepared by adding 20 μL of the unknown sample and 20 μL of substrate buffer. The ACE-I inhibitor captopril© was used as a positive control at the same concentration and following the same procedure as the samples in the inhibitor wells. The enzymatic reaction was started by adding 20 μL of enzyme working solution to each inhibitor and negative control wells. The plate was covered and incubated at 37 °C. After 1 h, 200 μL of indicator working solution was added to each well and the microwell plate was further incubated at room temperature for 10 min. The absorbance of the reaction was measured with a microplate reader (FLUOstar Omega, BMG Labtech, Offenburg, Germany) at 450 nm. The ACE-I inhibition percentage for each sample was calculated using Equation (1):

% Inhibition_ACE-I_ = [(A_blank1_ − A_inhibitor_)/(A_blank1_ − A_blank2_)] × 100. (1)

#### 3.6.2. Renin Inhibition Assay

The renin inhibition assay was performed using an enzymatic kit (Cayman Chemical Company) and following the manufacturer’s recommendations. *Ulva* sp. proteins, hydrolysates and the positive control (Z-Arg-Arg-Pro-Phe-His-Sta-Ile-His-Lys-(Boc)-OMe) were dissolved in DMSO at a concentration of 1 mg/mL. The assay started by transferring 10 μL of samples and control to a microplate, followed by the addition of 20 μL renin substrate, 150 μL assay buffer and 10 μL renin. The plates were incubated at 37 °C for 15 min and the fluorescence of the reaction was read at excitation wavelengths of 340 nm and emission wavelengths of 500 nm in a microplate reader (FLUOstar Omega, BMG Labtech, Offenburg, Germany). The percentage of renin inhibition was calculated using Equation (2):

% Inhibition_renin_ = [(100% Initial activity − Inhibitor)/100% Initial activity] × 100. (2)

### 3.7. Identification of Peptides by Mass Spectrometry

ACE-I inhibitory and renin inhibitory fractions were characterised using liquid chromatography and tandem mass spectrometry (LC-MS/MS). The samples were re-suspended in 50 μL in 2% ACN containing 0.1% TFA and 5 µL were injected into a Nano-LC Ultra 1D Plus system (Eksigent AB Sciex, Dublin, CA, USA) and pre-concentrated using a trap column (C18-CL Eksigent AB Sciex, 3 µm, 350 μm × 0.5 mm) for 5 min using 0.1% TFA at a flow rate of 3 µL/min. The samples were loaded onto an analytical column (C18-CL Nikkyo, 3 µm, 75 μm × 12 cm) equilibrated in 0.1% FA in water. Elution was carried out using a linear gradient from 5% to 35% of solvent B in A for 45 min (solvent A: 0.1% FA in water; solvent B: ACN containing 0.1% FA) at a flow rate of 0.3 μL/min. The peptides were analysed using nanoESI qQTOF (nanoelectrospray ionization in a quadrupole/time-of-flight TripleTOF 5600+ system from AB Sciex Instruments (Framingham, MA, USA)). The sample was ionised by applying 2.8 kV to the spray emitter and the analyses were carried out in a data-dependent mode. MS1 scans were acquired from 350 to 1250 *m*/*z* for 250 ms, the quadrupole resolution was set to ‘UNIT’ for MS2 experiments and the scans were acquired from 100 to 1500 *m*/*z* for 50 ms in ‘high sensitivity’ mode. The switch criteria used were charge (1+ to 5+), minimum intensity and 70 counts per second (cps). Up to 25 ions were selected for fragmentation after each survey scan and the dynamic exclusion was set to 15 s.

The data from LC-MS/MS were further processed using ProteinPilot version 5.0 search engine (AB Sciex, Framingham, MA, USA) using the default parameters to generate the peak list directly from the 5600 TripleTOF 5600+ files. The Paragon algorithm (Shilov et al. [49]) of ProteinPilot version 5.0 was used to search in Uniprot and NCBI_GreenAlgae databases, selecting no enzyme specificity, no taxonomy restriction and with the search effort of the software set to “Thorough”.

### 3.8. In Silico Analyses

The bitterness of the characterised peptides was estimated by the “Q rule” proposed by Ney [34]. This estimation method is based on the calculation of a Q-value for each peptide on the basis of its amino acid composition and the solubility data of each of the individual amino acids of the peptide sequences.

Furthermore, characterised peptides from *Ulva* sp. were assessed for potential cleavage by GI tract enzymes including pepsin pH 1.3 and pH > 2.0 (EC 3.4.23.1), trypsin (EC 3.4.21.4) and chymotrypsin (EC 3.4.21.1) using the ExPASy PeptideCutter tool (http://web.expasy.org/peptide_cutter/). The novel peptide sequences generated after the GI in silico digestion were also compared against the BIOPEP database (http://www.uwm.edu.pl/biochemia/index.php/pl/biopep) to identify the previously reported active sequences [32]. The allergenicity of the novel sequences after the GI in silico digestion was predicted using AllerTOP version 2.0 (http://www.ddg-pharmfac.net/AllerTOP/index.html). The toxicity of all the peptides was scored in ToxinPred (http://crdd.osdd.net/raghava/toxinpred/) using a virtual scanning method (VSM) Swiss-Prot based and a SVM threshold of 0.0 [50]. The potential biological activity of all the peptides was predicted using the scores calculated in PeptideRanker (http://distilldeep.ucd.ie/PeptideRanker/) [51].

### 3.9. Statistical Analyses

All the statistical analyses were performed using SPSS version 24.0. The biological activities in vitro of the different protein, hydrolysate and fractions were analysed using a univariate general linear model and LSD post hoc tests. In all the cases the criterion for statistical significance was *P* < 0.05.

## 4. Conclusions

This study shows the great potential of *Ulva* sp. protein hydrolysates generated using the food-grade enzyme papain as a source of bioactive peptides. The hydrolysates and fractions generated had low renin and high ACE-I inhibitory activities in vitro. The peptides of the most active hydrolysates were fully identified and in silico tools including ExPASy PeptideCutter, ToxinPred and AllerTOP were used to predict the resistance of the peptides to gastrointestinal enzymatic digestion, toxicity and allergenicity of the peptides, respectively. Using this mixed in vitro‒in silico approach, 48 novel peptides were identified and 86 novel amino acid sequences were generated after the in silico gastrointestinal simulation. Although the peptides were predicted to be non-toxic, several sequences were predicted to be probably allergenic. Further in vivo studies will be needed to confirm the antihypertensive activity of the hydrolysate and the safety of the identified bioactive peptides.

## Figures and Tables

**Table 1:** Peptides identified by HPLC-MS/MS from Fr41‒44 collected by RP-HPLC of *Ulva* sp. protein hydrolysates with papain. The Q-values are an estimation of the bitterness of the peptides and are calculated and summarised in the last column.

Samples	Amino Acid Sequences	Modifications	Observed MW	Calculated MW	Observed *m*/*z*	Theoretical *m*/*z*	Charge (+)	Database	Accessions	Q-Values
**Fr41**	TGGSLHAA		712.41	712.35	357.21	357.18	2	NCBInr	XP_005851095.1; EFN58993.1	607.5
	VVPKAAPPPN		988.76	988.57	495.39	495.29	2	NCBInr	XP_005843189.1; EFN51087.1	1343
	ATKPAN		600.41	600.32	301.21	301.17	2	NCBInr	XP_001696486.1; XP_001696477.1	1001.67
	SGAASASGAA		748.40	748.34	375.21	375.17	2	NCBInr	RRRRRKXZ51346.1	377
	HSAVFAAS		788.51	788.38	395.26	395.20	2	NCBInr	GAX77766.1	883.75
	NGNAASPGQPPLEVH		1486.84	1486.72	744.43	744.37	2	NCBInr	RRRRRJAC80986.1	960
	HQWGGAPQH		1016.69	1016.46	509.35	509.24	2	NCBInr	XP_002500034.1; ACO61292.1	794.44
	KFKGMNHINDIEKFK		1847.96	1847.97	616.99	617.00	3	UniProt	sp|B9DRW5	1304
	GAVHAMVDTAKIHKGKK		1789.95	1790.00	597.66	597.67	3	UniProt	RRRRRsp|Q3TC72	1048.23
**Fr42**	AGGPNQPPN		850.48	850.39	426.25	426.20	2	NCBInr	XP_001694488.1; EDP02483.1	941.11
	AANITVPAAN		940.58	940.50	471.30	471.26	2	NCBInr	XP_002957564.1; EFJ41334.1	1062
	DSLSAIGGAPDG		1058.45	1058.49	530.23	530.25	2	NCBInr	JAC65094.1	885.83
	SAGVLPWK		856.50	856.48	429.26	429.25	2	NCBInr	KXZ46138.1	1500
	DGLIDGL		694.35	701.36	695.36	702.37	1	UniProt	tr|A0A1C9C803|A0A1C9C803_9FLOR	1270
	GDLIDVA		694.35	701.36	695.36	702.37	1	UniProt	tr|M1VAH3|M1VAH3_CYAM1	1270
	NQVTNLIVA		970.46	970.54	486.24	486.28	2	UniProt	tr|A0A1G4NVL3|A0A1G4NVL3_9FLOR	1091.11
	GSASGAFVY		857.46	857.39	429.74	429.70	2	UniProt	tr|M1V5F7|M1V5F7_CYAM1	972.22
**Fr43**	HGPPPPSPYRSAAGRAAL		1800.90	1800.94	601.31	601.32	3	NCBInr	XP_005846003.1; EFN53901.1	1297.22
	EAEPAEAA		786.37	786.34	394.19	394.18	2	NCBInr	XP_005852249.1; EFN60147.1	898.75
	SIAGVAAFIG		904.48	904.50	453.25	453.26	2	NCBInr	JAC64130.1	1251
	YAAKMRK		866.54	866.48	434.28	434.25	2	NCBInr	XP_007512502.1; CCO17102.1	1337.14
	DDLKGTF		794.38	794.38	398.20	398.20	2	NCBInr	XP_003058074.1; EEH58025.1	1155.71
	GGVYGTSAR		866.50	866.42	434.26	434.22	2	NCBInr	XP_001700029.1; EDP07725.1	722.22
	GSECMWFAS		1016.33	1016.37	509.17	509.19	2	NCBInr	XP_001693035.1; EDP03604.1	923.33
	AGFSYFGESS		1050.45	1050.43	526.23	526.22	2	NCBInr	XP_002951597.1; EFJ47408.1	957
	DLLALRELDVACN		1443.77	1443.74	722.89	722.88	2	NCBInr	JAT73014.1	1167.69
	NGGDLPGAL		812.42	812.40	407.22	407.21	2	NCBInr	XP_003055625.1; EEH60877.1	968.89
	ALLQQQAQMAAALPLPP		1759.90	1759.97	587.64	587.66	3	NCBInr	XP_005847720.1; EFN55618.1	1299.41
	LTPCAVPE		828.43	828.41	415.22	415.21	2	NCBInr	KXZ44026.1	1383.75
	ERTGRVAM		918.48	918.47	460.25	460.24	2	NCBInr	KXZ43890.1	771.25
	LNCALK		660.43	660.36	331.22	331.19	2	NCBInr	XP_001416982.1; ABO95275.1	1176.67
	GAAPTPPSPPPATKPSTPPKPPT		2190.15	2190.17	731.06	731.06	3	NCBInr	XP_002958348.1; EFJ40570.1	1558.69
	IECCLLFALV		1122.55	1122.58	562.28	562.30	2	NCBInr	GAX82307.1	1585
	PVGCLPK		712.40	712.39	357.21	357.20	2	NCBInr	JAC75907.1	1550
	DEDESSFGK		1012.47	1012.40	507.24	507.21	2	UniProt	tr|R7QMS5|R7QMS5_CHOCR	712.22
	GASPVTFVFT		1024.49	1024.52	513.25	513.27	2	UniProt	tr|M1VHB3|M1VHB3_CYAM1	1295
	AGDLGAYG		722.39	722.32	362.20	362.17	2	UniProt	tr|A0A1X6NU59|A0A1X6NU59_PORUM	911.25
	RSARVRVGSTAT		1259.69	1259.71	630.85	630.86	2	UniProt	tr|A0A1X6NKL7|A0A1X6NKL7_PORUM	665.83
	INNNKIITNL	Deamidated (N)@2	1156.65	1156.65	579.33	579.33	2	UniProt	tr|A0A1Z1MP80|A0A1Z1MP80_9FLOR	1323
	DAVEIWRVK		1114.58	1114.61	558.30	558.31	2	UniProt	tr|R7QMS0|R7QMS0_CHOCR	1488.89
**Fr44**	DEVIPGAL		812.43	812.43	407.22	407.22	2	NCBInr	GAX84002.1	1440
	DATFCGDLDDA		1141.54	1141.42	571.78	571.72	2	NCBInr	XP_001697003.1; EDP00695.1	830
	PKPPALCN		838.47	838.44	420.24	420.23	2	NCBInr	KXZ44308.1	1562.5
	PPNPPNPPN		942.56	942.46	472.29	472.24	2	NCBInr	XP_002955492.1; EFJ43345.1	1743.33
	TPALVSQLH		964.50	964.53	483.26	483.27	2	NCBInr	XP_011396301.1; KFM23431.1	1195.56
	TMSDRFL		868.51	868.41	435.26	435.21	2	NCBInr	XP_002499460.1; ACO60718.1	1160
	AAGAAPLL		682.44	682.40	342.23	342.21	2	NCBInr	AAW51128.1	1297.5
	LIKELDSN		930.62	930.50	466.32	466.26	2	UniProt	tr|A0A1Z1MG56|A0A1Z1MG56_9FLOR	1303.75

**Table 2:** Bioactive peptides generated after the in silico gastrointestinal digestion of the identified peptide from *UIva* sp. already available in the scientific literature. The data were attained from the BIOPEP database (http://www.uwm.edu.pl/biochemia/index.php/pl/biopep) on 14 February 2019.

Amino Acid Sequences	Bioactivity In Vitro *	Reference
AA	ACE-I inhibitor	Cushman et al. [37]
AG	ACE-I inhibitor	Cheung, Wang, Ondetti, Sabo and Cushman [29]
AS	DPP IV inhibitor	Lan et al. [38]
DG	ACE-I inhibitor	Meisel, Walsh, Murray and Fitzgerald [7]
GA	ACE-I inhibitor	Cheung, Wang, Ondetti, Sabo and Cushman [29]
GD	ACE-I inhibitor	Cheung, Wang, Ondetti, Sabo and Cushman [29]
GGV	HMG-CoA reductase inhibitor	Soares, Mendonça, de Castro, Menezes and Arêas [36]
GK	ACE-I inhibitor	Cheung, Wang, Ondetti, Sabo and Cushman [29]
GM	ACE-I inhibitor	Cheung, Wang, Ondetti, Sabo and Cushman [29]
IG	ACE-I inhibitor	Cheung, Wang, Ondetti, Sabo and Cushman [29]
IH	DPP IV inhibitor	Lan, Ito, Ohno, Motoyama, Ito and Kawarasaki [38]
NH	DPP IV inhibitor	Lan, Ito, Ohno, Motoyama, Ito and Kawarasaki [38]
PK	DPP IV inhibitor	Lan, Ito, Ohno, Motoyama, Ito and Kawarasaki [38]
TM	DPP IV inhibitor	Lan, Ito, Ohno, Motoyama, Ito and Kawarasaki [38]
VK	DPP IV inhibitor	Lan, Ito, Ohno, Motoyama, Ito and Kawarasaki [38]
	ACE-I inhibitor	Wang and De Mejia [39]
VR	DPP IV inhibitor	Nongonierma and FitzGerald [40]
	ACE-I inhibitor	Gómez-Ruiz et al. [41]

* Abbreviations in the table correspond to: angiotensin-I-converting enzyme (ACE-I), dipeptidyl peptidase IV inhibitor (DPP IV inhibitor), 3-hydroxy-3-methylglutaryl-coenzyme A reductase inhibitor (HMG-CoA reductase inhibitor).

**Table 3:** Peptides identified as having the potential to cause allergy obtained after in silico gastrointestinal digestion and assessment using online tools ToxinPred and Peptideranker. The toxicological, bioactivity scores and the nearest protein identified in different databases are also summarised for each peptide.

Amino Acid Sequence	MW (Da)	SVM Scores ^a^	PeptideRanker Scores ^b^	Nearest Protein
AANITVPAAN	940.4978	−1.3	0.144457	NCBI GI number 2154734
AAPPPN	565.627	−0.46	0.792864	NCBI GI number 33323477
AGD	261.235	−0.8	0.283992	UniProtKB accession number O01949
AGGPNQPPN	850.3933	−0.72	0.621643	NCBI GI number 33323477
CGD	293.295	−0.79	0.646691	NCBI GI number 102834
DD	248.192	−0.79	0.098852	UniProtKB accession number Q17282
DDA	319.271	−0.8	0.127455	UniProtKB accession number O01949
DEVIPGA	699.759	−0.45	0.185675	NCBI GI number 543491
EVH	383.404	−0.82	0.0380427	NCBI GI number 21465915
GASPVT	530.579	−0.97	0.247962	NCBI GI number 2500822
GESS	378.339	−0.76	0.0716116	NCBI GI number 32363197
GGAPQH	565.586	−0.86	0.388533	UniProtKB accession number P86254
GSASGA	448.433	−0.93	0.250858	NCBI GI number 543482
GSECM	525.592	−0.42	0.554283	NCBI GI number 162927
IDVA	416.475	−0.84	0.0871288	NCBI GI number 539716
IECC	466.568	−0.25	0.448357	NCBI GI number 14285595
IITN	459.543	−0.84	0.106012	UniProtKB accession number Q93YG7
IK	259.349	−0.8	0.0974139	NCBI GI number 157829757
INNNK	601.66	−0.81	0.0938778	NCBI GI number 1083651
NGNAASPGQPPL	1122.203	−0.78	0.505873	NCBI GI number 33323477
NQVTN	574.591	−1.01	0.0559664	UniProtKB accession number P00709
SAIGGAPDG	743.771	−0.67	0.397354	NCBI GI number 285005077
SAR	332.36	−0.81	0.278711	NCBI GI number 81890324
TGR	332.36	−0.8	0.276659	UniProtKB accession number Q7M1M4
TPAL	400.475	−0.8	0.342157	NCBI GI number 18203509
VAM	319.419	−0.8	0.264259	NCBI GI number 9072
VSQ	332.357	−0.83	0.0492778	NCBI GI number 2147092
VVPK	441.571	−0.88	0.0810705	NCBI GI number 2133755

^a^ SVM scores obtained from in silico analyses using ToxinPred (http://crdd.osdd.net/raghava/toxinpred/). Data retrieved on 15 February 2019. ^b^ Biological activity scores obtained from in silico analyses using Peptideranker (http://distilldeep.ucd.ie/PeptideRanker/). Data retrieved on 14 February 2019.

**Table 4:** Probably non-allergenic peptides obtained after in silico gastrointestinal digestion. The toxicological, bioactivity scores and the nearest protein identified in different databases are also summarised for each peptide.

Amino Acid Sequence	MW (Da)	SVM Scores ^a^	PeptideRanker Scores ^b^	Nearest Protein
AAAL	344.411	−0.84	0.28869	UniProtKB accession number A9UGV6
AAGAAP	456.499	−1.01	0.352249	UniProtKB accession number Q7M1V0
AAK	288.347	−0.77	0.113614	UniProtKB accession number P27807
AAL	273.332	−0.84	0.310569	UniProtKB accession number A9UGV6
AAS	247.251	−0.82	0.123531	UniProtKB accession number A2WQG7
AM	220.287	−0.8	0.74549	UniProtKB accession number Q8IWT0
ATKPAN	600.32312	−0.88	0.129647	UniProtKB accession number Q8WYQ3
CN	235.258	−0.8	0.63423	UniProtKB accession number P01052
DAT	305.288	−0.81	0.0954274	UniProtKB accession number Q9NNX6
DAVEI	545.59	−0.93	0.0603561	UniProtKB accession number Q9T2R4
DEDESS	680.58	−0.78	0.0365686	UniProtKB accession number Q9S8K0
DS	220.182	−0.8	0.0878061	UniProtKB accession number O75366
DSN	334.286	−0.78	0.104772	UniProtKB accession number P31358
DVACN	520.558	−0.68	0.203726	UniProtKB accession number Q7M2H1
EAEPAEAA	394.193	−0.99	0.0830328	UniProtKB accession number Q9S8F6
ER	303.318	−0.8	0.0704548	UniProtKB accession number Q9T2R8
GAAPTPPSPPPATKPSTPPKPPT	731.0588	−0.69	0.378489	UniProtKB accession number Q9S8M0
GAVH	382.42	−0.77	0.173142	UniProtKB accession number Q9S8I6
GPPPPSP	647.729	−0.11	0.870562	UniProtKB accession number Q7M1V1
GTF	323.349	−0.8	0.84905	UniProtKB accession number P04234
GTSAR	490.517	−0.89	0.151592	UniProtKB accession number Q7M282
IDG	303.315	−0.78	0.288785	UniProtKB accession number P86001
INDIEK	730.816	−0.97	0.0849714	UniProtKB accession number P86006
IVA	301.386	−0.79	0.0757333	UniProtKB accession number Q9S8N5
NCA	306.337	−0.77	0.425512	UniProtKB accession number A6N1B4
NGGDL	474.471	−0.76	0.567577	UniProtKB accession number P55857
PGA	243.263	−0.81	0.674335	UniProtKB accession number Q9S906
PKPPAL	621.778	−0.7	0.715386	UniProtKB accession number Q7M1U2
PLPP	422.525	−1.04	0.876691	UniProtKB accession number Q7M1V1
PPNPPNPPN	942.455933	−0.4	0.855282	UniProtKB accession number Q9S8M0
PVGCL	487.615	−0.63	0.734232	UniProtKB accession number P83184
QQQAQM	732.809	−0.92	0.216075	UniProtKB accession number Q9UL45
SAAGR	460.49	−0.85	0.341095	UniProtKB accession number P80825
SAGVL	445.516	−0.99	0.38464	UniProtKB accession number A9UGV7
SAV	275.305	−0.82	0.0814653	UniProtKB accession number Q09Y74
SDR	376.37	−0.83	0.242439	UniProtKB accession number Q9BXJ1
SGAASASGAA	748.335144	−1.17	0.2792	UniProtKB accession number Q7M1V0
SIAGVAA	587.674	−1.28	0.134975	UniProtKB accession number A9UGV6
TGGS	320.302	−0.77	0.17105	UniProtKB accession number Q15517
TPCAVPE	715.819	−0.88	0.224438	UniProtKB accession number Q9T2Q0
VDTAK	532.594	−0.34	0.0513549	UniProtKB accession number Q9T2R4
VGSTAT	534.567	−1.03	0.0691571	UniProtKB accession number Q10ST8

^a^ SVM scores obtained from in silico analyses using ToxinPred (http://crdd.osdd.net/raghava/toxinpred/). Data retrieved on 15 February 2019. ^b^ Biological activity scores from in silico analyses using Peptideranker (http://distilldeep.ucd.ie/PeptideRanker/). Data retrieved on 14 February 2019.
